# Ultrasound aspects and risk factors associated with urogenital schistosomiasis among primary school children in Mali

**DOI:** 10.1186/s40249-023-01071-6

**Published:** 2023-04-20

**Authors:** Privat Agniwo, Bakary Sidibé, Assitan Diakité, Safiatou Doumbo Niaré, Hassim Guindo, Ahristode Akplogan, Moudachirou Ibikounlé, Jérôme Boissier, Abdoulaye Dabo

**Affiliations:** 1 Department of Epidemiology of Infectious Diseases, Faculty of Pharmacy, IRl 3189 (USTTB/UCAD/UGB/CNRST/CNRS), University of Sciences, Techniques and Technologies of Bamako, Bamako, Mali; 2 Interactions Hôtes-Pathogènes-Environnements (IHPE), Univ. Montpellier, CNRS, Ifremer, Univ. Perpignan Via Domitia, Perpignan, France; 3 Centre de Recherche pour la lutte contre les Maladies Infectieuses Tropicales (CReMIT/TIDRC), University of Abomey-Calavi, Abomey Calavi, Benin

**Keywords:** *Schistosoma haematobium*, Morbidity, Prevalence, Risk factor, Mali

## Abstract

**Background:**

Urogenital schistosomiasis is endemic in Mali and is a major cause of serious morbidity in large parts of the world. This disease is responsible for many socio-economic and public health issues. The aim of this study was to investigate the impact of the disease on morbidity and to describe demographic and socioeconomic factors in relation to the status of children with urogenital schistosomiasis in Mali.

**Methods:**

We conducted a cross-sectional study in November 2021 of 971 children aged 6 to 14 years selected at random from six schools in three districts in the Kayes Region of Mali. Demographic and socioeconomic data were collected on survey forms. Clinical data were collected following a medical consultation. Hematuria was systematically searched for through the use of strips. The search for *Schistosoma haematobium* eggs in urine was done via the filtration method. The urinary tract was examined by ultrasound. Associations between each of these variables and disease infection were tested using multivariate logistic regression.

**Results:**

The overall prevalence of urinary schistosomiasis detected was 50.2%. The average intensity of infection was 36 eggs/10 ml of urine. The associated risk factors for urogenital schistosomiasis showed that children who bathed, used the river/pond as a domestic water source, and who habitually urinated in the river/pond were more affected (*P* < 0.05). Children with farming parents were most affected (*P* = 0.032). The collection of clinical signs revealed that boys had more pollakiuria (58.6%) and dysuria (46.4%) than girls. Ultrasound data showed that focal lesion rates were recorded in all villages with the lowest rate in Diakalel (56.1%). Ultrasound and parasitological findings showed that irregularity and thickening were strongly associated with urinary schistosomiasis (*P* < 0.0001).

**Conclusions:**

*Schistosoma haematobium* infection was still endemic in the study site despite more than a decade of mass treatment with praziquantel. However, the high percentage of symptoms associated with high intensity reinforces the idea that further studies in terms of schistosomiasis-related morbidity are still needed.

## Background

Schistosomiasis is a neglected tropical disease (NTD) caused by the dioecious blood fluke from the genus *Schistosoma* [1]. Human schistosomiasis is one of the most widespread parasitic infections in the world and threatens the economy and public health systems in endemic countries. This disease is also the most common waterborne disease among rural populations [2, 3]. According to World Health Organization (WHO) estimates, in 2021, chemoprevention against schistosomiasis was needed in 51 countries for a total of 251.4 million people, including 136 million school-age children [4], with sub-Saharan Africa and parts of Asia reporting the highest endemicity. Sub-Saharan Africa accounts for 90% of infections [5].

Urogenital schistosomiasis is caused by *Schistosoma haematobium*. The disease is the second most prevalent tropical disease after malaria, is a leading cause of severe morbidity in many places around the world and is a disease that heavily impacts socio-economic and public health systems, particularly in rural areas of developing countries [6–8].

In Mali, urogenital schistosomiasis due to *S. haematobium* and intestinal and hepatic schistosomiasis due to *S. mansoni* are both endemic [9] and occur around large hydroelectric facilities, small reservoirs, ponds, and rivers. While the former is widely distributed throughout the country, the latter is mainly focused on the irrigated rice-growing area of the Office du Niger in the Segou region. Rural and suburban areas are more exposed to the parasite than urban areas [10, 11].

Several studies have demonstrated the association between the epidemiology, prevalence, and risk factors of the disease. However, there are mixed views [12–15].

According to WHO recommendations, ultrasound is an excellent method for obtaining information about the changes that occur on the internal organs after schistosome infection [16]. *S. haematobium* can cause lesions of the genital tract. The prevalence and severity of pathological changes detected by ultrasound correlate with the intensity of infection, as measured by the frequency and quantity of eggs excreted in the urine. In most endemic areas, peak morbidity is observed in children aged 7–14 years [16]. In urinary schistosomiasis, the adult worms live mainly in the venous plexus of the urinary bladder. Morbidity is caused by egg deposition around the urinary tract, causing inflammation and lesions; thus, the pathology is mainly found in the urinary bladder, ureters, and kidneys [2].

The most common lesions are irregularities of the bladder wall, a distorted bladder shape, and wall thickening; bladder masses may be also present. Lesions in the upper urinary tract, such as ureter dilatation and hydronephrosis, are less frequent but usually more severe, indicating a higher level of pathology [17, 18].

The mass treatment strategy targeting school children as a means of controlling schistosomiasis has been in place for over a decade. The objective of this work is to study the impact of prevention efforts on morbidity and to describe any changes in demographic and socioeconomic factors related to the disease.

## Methods

### Study population and data collection

**Fig. 1:**
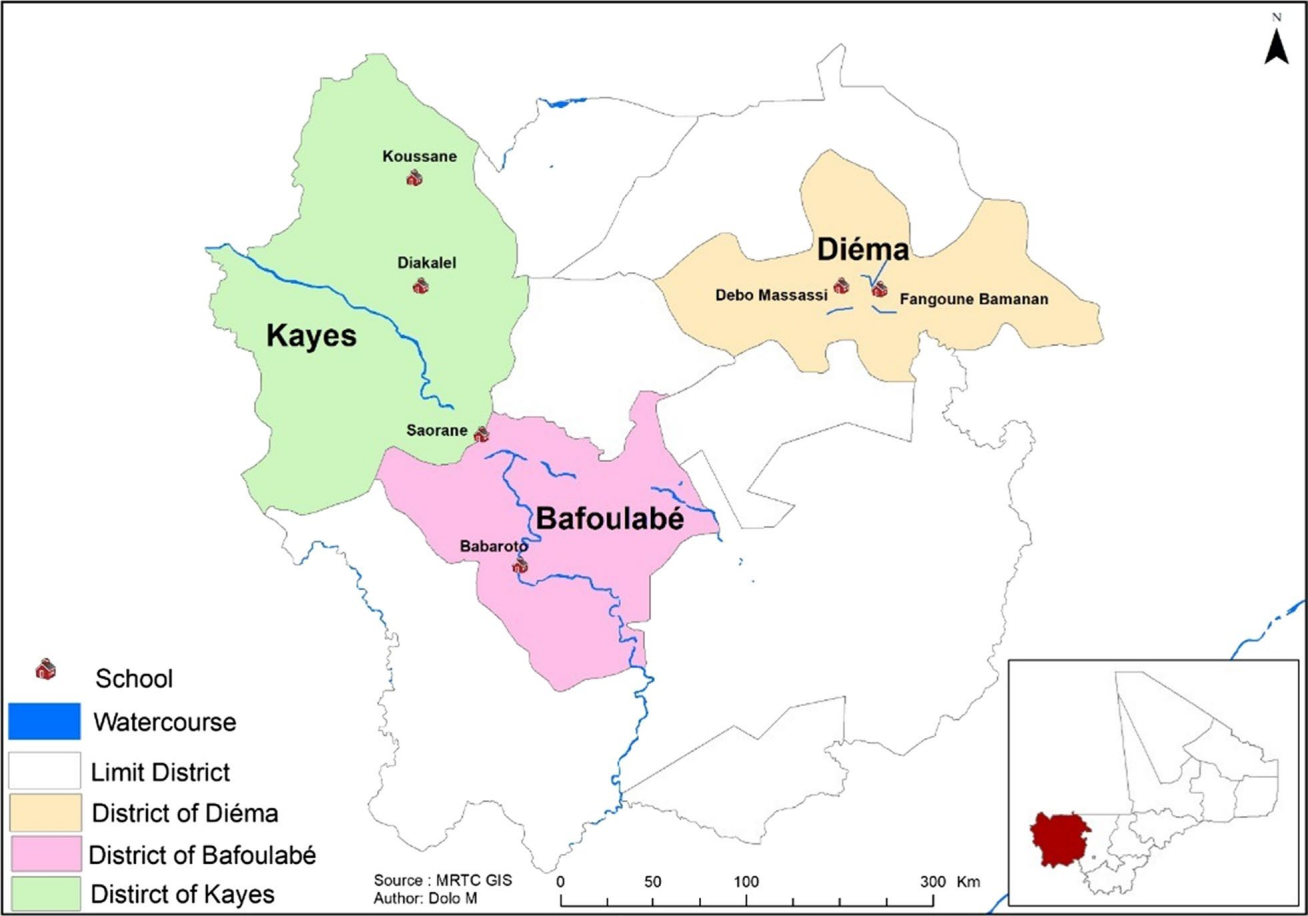
Localization of the study sites in the Kayes region in Mali (West Africa) (Source: MRTC – GIS; Author, Dolo M)

The study took place in six villages endemic for *S. haematobium* in the Kayes Region, western Mali [19]. This region is divided into three sanitary districts: Kayes, Bafoulabé and Diéma. The distances between the districts ranged from 20 to 45 km. The study villages were chosen according to their proximity to the water points (ponds in Diéma, Senegal River and its tributaries in Kayes and Bafoulabé). Agriculture and livestock are the population’s two main economic activities [20]. Two districts (Bafoulabé and Kayes) belong to the North-Soudanian climatic zone with two major seasons: the wet season from May–June to October with its beginning and end marked by torrential rains and thunderstorms, and the dry season from November to April–May. The mean annual rainfall is up to 1000 mm, which occurs mainly during the period from July to September. Diéma, located further north, has a Sahelian climate and two distinct seasons: a wet season from July to September–October and a dry season that spans the rest of the year. The annual rainfall is about 600 to 800 mm [20]. Two villages were selected in each district: Diakalèl and Koussané in Kayes; Babaroto and Saorané in Bafoulabé and Fangouné Bamanan and Débo Massassi in the Diéma district (Fig. 1).

### Type and period of the study

The study was an observational cross-sectional study that took place from 7 to 21 November 2021. The studied population consisted of children from 6 to 14 years old, who attending in one of the six selected primary schools and who agreed to participate in the study. The minimum sample size was calculated based on the previous prevalence of the disease obtained in each school using the Schwartz formula, considering a 10% refusal rate and sampling errors. (*National Schistosomiasis and Geohelminth Control Program, report 2015*).

### Data collection procedures (socio-demographic, socio-economic, paraclinical and clinical data collection procedures)

A total of 1087 children were sampled. Children were randomly selected from the list of children in each class until the required number of samples were obtained. Sociodemographic data, including village, gender, age, were obtained using a structured questionnaire. Socioeconomic information was collected through questionnaires administered by a sociologist. The questions were close ended to facilitate the children's responses. For the parents' level of education (yes or no), type of toilet (modern or traditional), frequency of use of streams (No: Never frequents the river; Yes: I frequent the river several times a week), type of water used (drilling, river, rain, drinking), and reason for frequenting streams (swimming, fetching water, taking a bath). The teachers helped us if the child had difficulties answering. Clinical signs (abdominal pain, pollakiuria, dysuria) were collected through questioning and physical examination by a general practitioner. Microscopic hematuria was determined using Hemastix test strips (Ref: AZA—TESTUR10, Siemens Medical Solutions Diagnostics). Data were recorded on survey forms and each child was given a distinct identifier.

#### Parasitological examination

All urine samples were collected between 10:00 am and 2:00 pm (the favorable period for the elimination of eggs in the urine) in the field, by trained laboratory technicians. From each subject, urine was collected in a properly labeled sample container. A filtration technique was used to analyze the samples. A total of 10 ml urine was taken from each sample flacon after mixing it. The mixed sample was filtered through a Whatman filter (CAT No. 1001-025, 25 mm) which was stained with a 3% ninhydrin solution. After drying, the filters were immersed into water and then examined under a microscope for characteristics of terminal spurred schistosome eggs [21]. The prevalence rate of schistosomiasis in the schools was defined as the number of positive individuals per school per total number of individuals examined per school and multiplied by 100. *S. haematobium* intensity was classified into three categories: (i) no eggs; (ii) slight (1–49 eggs per 10 ml of urine); and (iii) heavy (≥ 50 eggs per 10 ml of urine).

#### Ultrasound examination

Ultrasound evaluations were carried out by a specialist with a portable ultrasound device, (Digital Ultrasonic Diagnostic Imaging System—Mindray, 15″ LCD display with tilt angle for better visualization, Lakeshore Burtchville, MI 480591-800-364-4942). All examinations were conducted by the same specialist at all sites. Several features were identified on ultrasound. The shape of the bladder was recorded, the detected lesions on the bladder wall were defined, and the degree of dilatation of the ureters and renal pelvis were measured, in accordance with current WHO guidelines [16]. The exact characterization of pathological changes was calculated using the global score as an index of the severity of morbidity and lesions. Children were asked to drink plenty of water before the ultrasound examination, which could only be conducted on a full bladder. Any marked abnormalities in the bladder, ureter and especially the renal pelvis, systematically prompted the resumption of ultrasound after urination to exclude the possibility of dilatation due to bladder and ureteral repletion. Urinary tract abnormalities were assessed based on the criteria defined by WHO. For successive analyses, children with at least one point on the WHO score were classified as having “urinary tract anomalies.” Bladder abnormalities were detected based on the shape and measurement of the bladder walls. A rectangular shape signified a normal bladder shape and corresponded to a score of (0), whereas bladder deformity corresponded to a rounded shape with a score of (1). Irregularity of the inner bladder wall with a thickening ≤ 5 mm was recorded as normal (score = 0), whereas a thickening > 5 mm was recorded as the presence of a lesion (focal, score = 1; multiple, score = 2). Posterior bladder wall thickness ≤ 5 mm was recorded as normal (score = 0) but posterior bladder wall > 5 mm was recorded as lesion (focal, score = 1; multiple, score = 2). A mass with local thickening ≤ 10 mm was normal (score = 0) in contrast to a mass with thickening > 10 mm was recorded as a mass (single, score = 1; multiple, score = 2). The polyp was defined based on wall growth with the presence being single or multiple. Dilation of each ureter and kidney was recorded separately. An absence of dilation corresponded to a score of (0), presence corresponded to a score of (3 = dilated; 4 = strongly dilated). Total scores were calculated based on the standard lesions observed to classify the likelihood of having schistosomiasis (≤ 1 = schistosomiasis unlikely; 2 = schistosomiasis likely; > 3 = schistosomiasis very likely) [16]. The sample size of children screened by ultrasound was 240, or 40 children per school.

### Statistical analysis

Socio-demographic, clinical and parasitological data were recorded on survey forms with identifiers for each child. The data was recorded in Excel. Calculations of prevalence, and intensity of infection were made using SPSS (IBM, version 23.0, Armonk,USA). Binomial analysis was performed on R software (Lucent Technologies, Jasmine Mountain, USA) to assess the relationship between clinical signs and urogenital schistosomiasis infection. Participants were divided into two age groups (6–10 and 11–14 years) for each sex. We performed statistical analyses to assess the relationship between parasitic infections and demographic, sociocultural, socioeconomic, clinical signs, and sonographic factors using a multivariate logistic regression. The differences in proportions were tested using the chi-square test or the exact Fisher test depending on the data. *P* values below 0.05 were considered significant.

## Results

### Socio-demographic characteristics of the paritcipants

A total of 971 children participated in the study with 38.5% (*n* = 374) girls and 61.5% (*n* = 597) boys. The highest participation was from Diakalèl (*n* = 251) and the lowest from Babaroto (*n* = 86) (Table 1). The 6–11 years age group was the most common (50.9%) (Table 1).

**Table 1:** Univariate analyses of selected environmental and socio-demographic variables

Socio-demographic variables	Total	*S. haematobium*		Egg load			
		Positive *n* (%)	*P*-value	Negative *n* (%)	Slight *n* (%)	Heavy *n* (%)	*P*-value
Sites (schools)			< 0.0001				< 0.0001
Baboroto	86	25 (29.1)		61 (70.9)	21 (24.4)	4 (4.7)	
Débo Massassi	224	67 (29.9)		157 (70.1)	58 (25.9)	9 (4.0)	
Diakalel	251	196 (78.1)		55 (21.9)	148 (59.0)	48 (19.1)	
Fangouné Bamanan	142	76 (53.5)		66 (46.5)	61 (43.0)	15 (10.6)	
Koussané	138	67 (48.6)		71 (51.4)	56 (40.6)	11 (8.0)	
Saorané	130	57 (43.8)		73 (56.2)	52 (40.0)	5 (3.8)	
Sex			0.13				0.86
Girls	374	197 (52.7)		177 (47.3)	152 (40.6)	45 (12.0)	
Boys	597	291 (48.7)		306 (51.3)	244 (40.9)	47 (7.9)	
Age (years)			0.13				0.58
6–11	494	239 (48.4)		255 (51.6)	185 (37.4)	54 (10.9)	
11–14	477	249 (52.2)		228 (47.8)	211 (44.2)	38 (8.0)	

*n* number; % percentage

### Prevalence and intensity of *S. haematobium* infection

The overall prevalence of urinary schistosomiasis was 50.2% with an average intensity of 36 eggs/10 ml of urine and the number of eggs varied from 1 to 1020 per sample. Infection varied significantly according to schools (78.1% in Diakalel vs 29.1% in Babaroto) (*P* = 0.0001). Variation of infection by gender and age was not significant (*P* = 0.13). In total, 9.5% of children were highly infected, the majority of which were from Diakalel (Table 1).

### Correlation between *S. haematobium* infection and socio-economic status and environmental factors

Socio-economic data collected show that almost all schools (99.6%) had a traditional toilet. Most children had various reasons for visiting pools or river (98.1%). The main reason for going was swimming (80.7%). Domestic water sources were variables, but the river was the main source of water (50.2%). Most children lived near the river/pond (94.4%) and used to urinate in the water source (92.5%). Concerning parents, agriculture was the primary activity (29.0%).

Multivariate analysis of socio-economic parameters associated with urogenital schistosomiasis showed that children with a modern toilet were all affected (*P* = 0.083) with a medium burden (75%) (*P* = *0.008*). Affected children indicated swimming as the main reason for river use and had high parasite loads (*P* < 0.0001). Prevalence and intensity of infection varied significantly with respect to the source of the domestic water supply. Those who collected water from the river were more affected (*P* < 0.0001) than those who didn’t. Most affected children usually urinated in the river/pond* (P* = 0.028*).* An analysis of the children's parents’ professions showed that those with farming parents were significantly more affected (*P* = 0.032) (Table 2).

**Table 2:** Multivariate logistic analyses showing correlation between *S. haematobium* infection and socio-economic status and environmental factors

Socio-economic factors	Total	*S. haematobium* infection		Egg load			*P*-value
		Positive	*P*-value	Negative	Slight	Heavy	
		*n* (%)		*n* (%)	*n* (%)	*n* (%)	
Type of toilet			0.083				0.008
Modern	4	4 (100.0)		0	3 (75.0)	1 (25.0)	
Traditionnel	967	483 (50.0)		483 (50.0)	393 (40.7)	90 (9.3)	
Frequentation of the river			0.41				0.24
Never frequents	18	10 (55.6)		8 (44.4)	10 (55.6)	0	
Several times a week	953	478 (50.2)		475 (49.8)	386 (40.5)	92 (9.7)	
Activity conducted at the river			< 0.0001				< 0.0001
Swim	784	348 (44.5)		435 (55.5)	288 (36.7)	61 (7.8)	
Search for water	18	11 (61.1)		7 (38.9)	10 (55.6)	1 (5.6)	
Bathing	151	118 (78.1)		33 (21.9)	88 (58.3)	30 (19.9)	
Home water source supply			< 0.0001				< 0.0001
River	487	175 (35.9)		312 (64.1)	151 (31.0)	24 (4.9)	
Drilling	96	71 (74.0)		25 (26.0)	56 (58.3)	15 (15.6)	
Rain	170	93 (54.7)		77 (45.3)	75 (44.1)	18 (10.6)	
Potable	218	149 (68.3)		69 (31.7)	114 (52.3)	35 (16.1)	
Distance from the house to the water source			0.54				0.12
Away	54	27 (50.0)		27 (50.0)	26 (48.1)	1 (1.9)	
Near	917	461 (50.3)		456 (49.7)	370 (40.3)	91 (9.9)	
Elimination of urine in water			0.028				0.10
No	73	45 (61.6)		28 (38.4)	38 (52.1)	7 (9.6)	
Yes	898	443 (49.3)		455 (50.7)	358 (39.9)	85 (9.5)	
Parent activity			0.032				0.14
Farmer	282	122 (43.3)		160 (56.7)	103 (36.5)	19 (6.7)	
Trader	69	42 (60.9)		27 (39.1)	33 (47.8)	9 (13.0)	
Official	37	17 (45.9)		20 (54.1)	14 (37.8)	3 (8.1)	
Fisherman	26	15 (57.7)		11 (42.3)	13 (50.0)	2 (7.7)	

*n*: number of samples;

### Clinical signs

Overall, an analysis of clinical signs in children showed that 46.1% of children had abdominal pain; 53.9%, pollakiuria; 39.6%, dysuria and 9.3% had hematuria. The majority of children who were positive for urogenital schistosomiasis also had urogenital hematuria (84.4%) (*P* = 0.0001) (Table 3).

**Table 3:** Clinical signs depending on the status of urinary schistosomiasis

Clinical signs	Total *n* (%)	Positive *n* (%)	Negative *n* (%)	Slight *n* (%)	Heavy *n* (%)	*P*-value
Vesical pain	448 (46.1)	218 (48.7)	230 (47.6)	170 (37.9)	48 (52.2)	0.18
Pollakiuria	524 (54.0)	246 (46.9)	278 (53.1)	197 (37.6)	49 (9.4)	0.16
Dysuria	385 (39.6)	202 (52.5)	183 (47.5)	159 (41.3)	43 (11.2)	0.27
Hematuria	90 (9.3)	76 (84.4)	14 (15.6)	43 (47.8)	33 (36.7)	0.0001

*n* number of samples

A multivariate analysis of clinical signs related to urinary schistosomiasis showed children with abdominal pain and dysuria were in the majority in Diakalèl and Débo Massassi, but pollakiuria was in the majority in Débo Massassi and hematuria in the majority in Diakalèl (Table 4). Only pollakiuria and dysuria varied significantly by sex, with boys more affected (*P* = 0.001) than girls, and the occurrence of abdominal pain was significantly higher in children aged 11–14 years than in younger children.

**Table 4:** Clinical signs stratified by study settings, sex, and age group

	Total	Clinical sign							
		Abdominal pain		Pollakiuria		Dysuria		Hematuria	
		*n* (%)	*P*-value	*n* (%)	*P*-value	*n* (%)	*P*-value	*n* (%)	*P*-value
Site			< 0.0001		< 0.0001		< 0.0001		< 0.0001
Baboroto	86	35 (9.1)		41 (7.8)		35 (9.1)		0	
Débo Massassi	224	127 (33.0)		195 (37.2)		127 (33.0)		7 (7.8)	
Diakalèl	251	127 (33.0)		93 (17.7)		127 (33.0)		61 (67.8)	
Fangouné Bamanan	142	28 (7.3)		30 (5.7)		28 (7.3)		18 (20.0)	
Koussané	138	41 (10.6)		100 (19.1)		41 (10.6)		3 (3.3)	
Saorané	130	27 (7.0)		65 (12.4)		27 (7.0)		1 (1.1)	
Sex			0.126		0.001		< 0.0001		0.45
Female	374	161 (43.0)		174 (46.5)		108 (28.9)		38 (10.2)	
Male	597	287 (48.1)		350 (58.6)		277 (46.4)		52 (8.7)	
Age (years)			0.009		0.061		0.43		0.468
6–11	494	209 (42.3)		283 (57.3)		194 (39.3)		41 (8.3)	
11–15	477	239 (50.1)		241 (50.5)		191 (40.0)		49 (10.3)	

*n* number of samples

### Ultrasonographic data and demographic variables

**Fig. 2:**
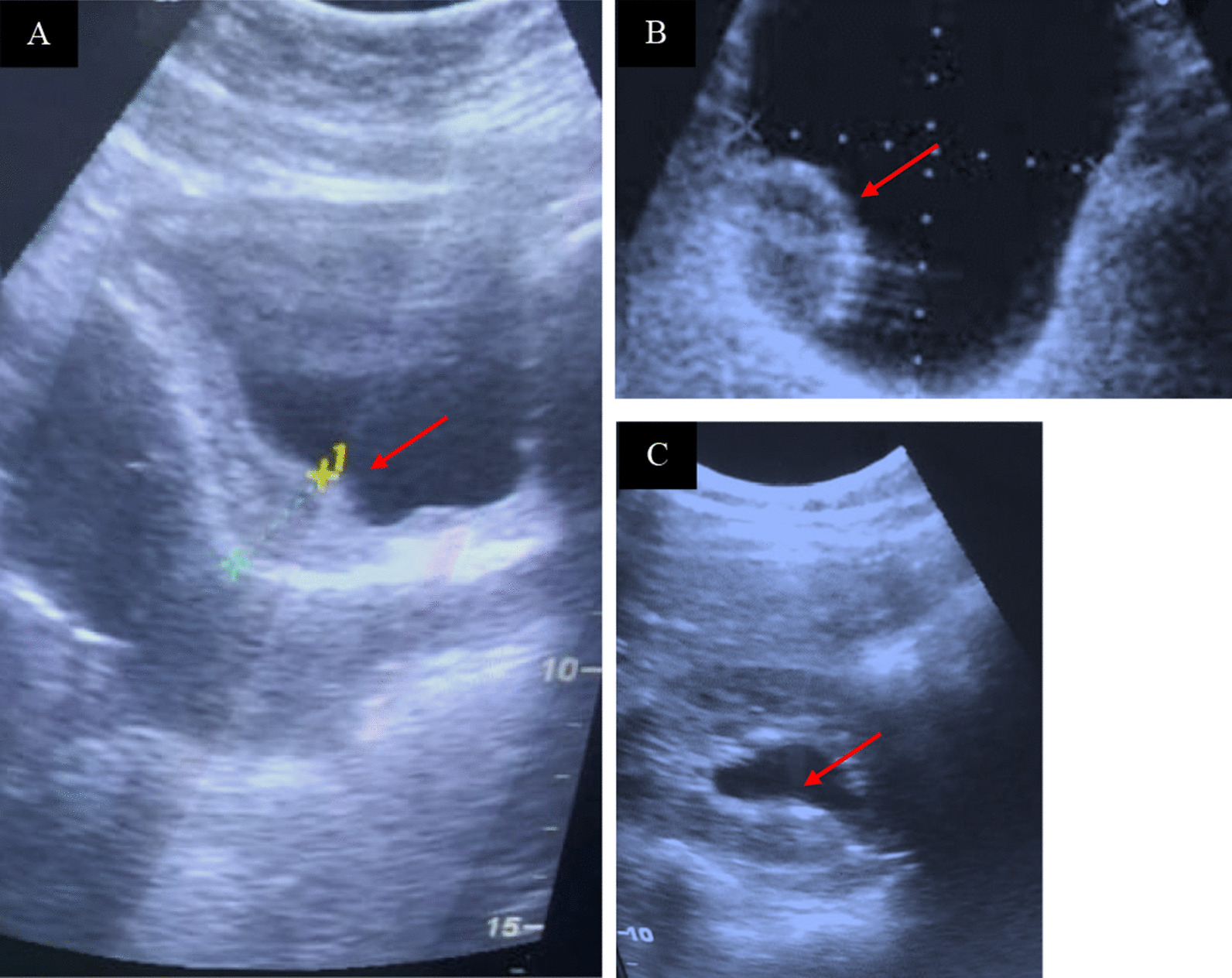
Ultrasound of children with urinary schistosomiasis **A** Irregular thickening of the bladder wall (11 mm); **B** thickening of the bladder wall associated with localized polyploidy; **C** nonobstructive dilatation of the left pyleic cavity

Among the 240 children who participated in the ultrasound examinations, the round shape was reported in only one child in Saorané. Focal lesion rates were recorded in all villages with a significantly low rate in Diakalèl (*P* < 0.0001). From these results, diffuse and focal thickenings were observed at significantly varying rates (*P* < 0.0001) with the highest rate of diffuse in Fangouné Bambana (32.5%) and focal in Débo Massassi (40.0%) (Fig. 2A, Table 5). A total of only three cases of single (2) and multiple (1) polyps were observed, and they occurred in Fangouné Bambana (100%) (Fig. 2B). The highly probable cases were recorded in Fangouné Bambana (54.5%), Débo Massassi (3.4%) and Diakalèl (9.1%), respectively *(P* < 0.0001). However, no statistical differences were associated with sex and age (Table 5).

**Table 5:** Distribution of ultrasound parameters of the bladder according to sites, sex and age in the Kayes region, Mali

	Total	Shape			Irregularity		Thickness			Mass			Polyp			Score			
		Normal	Round	*P*-value	Focal lesion	*P*-value	Diffuse	Focal	*P*-value	Multiple	Simple	*P*-value	Multiple	Simple	*P*-value	Unlikely	Probable	Very likely	*P*-value
*Site*				0.41		< 0.0001			< 0.001			0.015			0.168				< 0.001
Baboroto	39	39 (100.0)	0		35 (89.7)		7 (17.9)	9 (23.1)		0	0		0	0		23 (59.0)	16 (41.0)	0	
Débo Massassi	40	40 (100.0)	0		32 (80.0)		15 (37.5)	16 (40.0)		3 (7.5)	2 (5.0)		0	0		10 (25.0)	26 (65.0)	4 (10.0)	
Diakalèl	41	41 (100.0)	0		23 (56.1)		15 (36.6)	7 (17.1)		2 (4.9)	0		1 (2.4)	0		20 (48.8)	20 (48.8)	1 (2.4)	
Fangouné Bamanan	40	40 (100.0)	0		36 (90.0)		22 (32.5)	13 (12.5)		5 (12.5)	2 (5.0)		1 (2.5)	2 (5.0)		9 (22.5)	25 (62.5)	6 (15.0)	
Koussané	40	40 (16.7)	0		37 (92.5)		8 (20.0)	8 (60.0)		0	0		0	0		24 (60.0)	16 (40.0)	0	
Saorané	40	39 (100.0)	1 (2.5)		30 (75.0)		13 (27.5)	11 (40.0)		0	0		0	0		16 (40.0)	24 (60.0)	0	
*Sex*				0.59		0.15			0.35			0.60			0.23				0.12
Female	99	99 (100.0)	0		76 (76.8)		33 (33.3)	22 (22.2)		3 (3.0)	1 (1.0)		2 (2.0)	1 (1.0)		48 (48.5)	49 (49.5)	2 (2.0)	
Male	141	140 (99.3)	1 (0.7)		117 (83.0)		47 (33.3)	42 (29.8)		7 (5.0)	3 (2.1)		0	1 (0.7)		54 (38.3)	78 (55.3)	9 (6.4)	
*Age (years)*				0.67		0.51			0.66			0.095			0.12				0.16
6–11	80	80 (100.0)	0		64 (80.0)		24 (30.0)	21 (26.3)		1 (1.3)	0		1 (1.3)	2 (2.5)		38 (47.5)	41 (53.8)	1 (6.3)	
11–15	160	159 (99.4)	1 (0.6)		129 (80.6)		56 (35.0)	43 (26.9)		9 (5.6)	4 (2.5)		1 (0.6)	0		64 (40.0)	86 (67.7)	10 (90.9)	

Ultrasound findings of the urethra and kidneys revealed a low rate of urethral dilatation. Three cases of proximal and distal dilated urethra were recorded in Débo Massassi (1) and Diakalèl (2).

However, one child had a strongly dilated urethra (Diakalèl) (*P* = 0.54) (Fig. 1C). No abnormalities were observed for the kidneys (Table 6).

**Table 6:** Distribution of ultrasound parameters of the urethra and kidneys depending on the sites, sex and age

	Total	Dilated	Urethra	*P-*value	Kidney
			Strongly dilated		No dilated
Site				0.54	
Baboroto	39	0	0		39 (100.0)
Débo Massassi	40	1 (2.5)	0		40 (100.0)
Diakalèl	41	1 (2.4)	1(2.4)		41 (100.0)
Fangouné Bamanan	40	0	0		40 (100.0)
Koussané	40	0	0		40 (100.0)
Saorané	40	0	0		40 (100.0)
Sex				0.16	
Female	99	2 (2.0)	0		99 (100.0)
Male	141	0	1 (0.7)		141 (100.0)
Age (years)				0.46	
6–11	80	0	0		80 (100.0)
11–15	160	2 (1.3)	1 (0.6)		160 (100.0)

Multivariate analyses between ultrasound signs and parasitological results showed that irregularity and thickness were strongly associated to urinary schistosomiasis (*P* < 0.0001). Most strong carriers had a focal lesion (18.1%) and diffuse thickening (27.5%) (*P* < 0.0001). The only child with a grossly dilated urethra was a medium carrier (2.6%) (Table 7).

**Table 7:** Multivariate analyses of ultrasound variables associated with infection and its density

	*S. haematobium*			Egg load			*P*-value
	Total	Positive	*P*-value	Negative	Slight	Heavy	
	*n*	*n* (%)		*n* (%)	*n* (%)	*n* (%)	
Shape			0.68				0.63
Normal	239	162 (67.8)		77 (32.2)	124 (51.9)	38 (15.9)	
Round	1	1 (100.0)		0	1 (100.0)	0	
Irregularity			< 0.0001				< 0.0001
No lesion	47	12 (25.5)		35 (74.5)	9 (19.1)	3 (6.4)	
Focal lesion	193	151 (78.2)		42 (21.8)	116 (60.1)	35 (18.1)	
Thickness			< 0.0001				< 0.0001
Diffuse	80	68 (85.0)		12 (15.0)	46 (57.5)	22 (27.5)	
Focal	64	55 (85.9)		9 (14.1)	45 (70.3)	10 (15.6)	
Normal	96	40 (41.7)		56 (58.3	34 (35.4)	6 (6.3)	
Mass			0.11				0.28
Multiple	10	9 (90.0)		1 (10.0)	6 (60.0)	3 (30.0)	
Simple	4	4 (100.0)		0	3 (75.0)	1 (25.0)	
Polyp			0.38				0.41
Multiple	2	2 (100.0)		0	1 (50.0)	1 (50.0)	
Simple	2	2 (100.0)		0	2 (100.0)	0	
Score			< 0.0001				< 0.0001
Unlikely	102	46 (45.1)		56 (54.9)	39 (38.2)	7 (6.9)	
Probable	127	107 (84.3)		20 (15.7)	79 (62.2)	28 (22.0)	
Very likely	11	10 (90.9)		1 (9.1)	7 (63.6)	3 (27.3)	
Score urethra			0.48				0.13
Not dilated	237	160 (67.5)		77 (32.5)	123 (51.9)	37 (15.6)	
Dilated	2	2 (100.0)		0	2 (100.0)	0	
Roughly dilated	1	1 (100.0)		0	0	1 (100.0)	

## Discussion

Our work demonstrated an average prevalence of 50.1% of *Schistosoma haematobium*. This prevalence is higher than that reported for the same region by Dabo et al., in 2017 in 12 sentinel sites (26.8%) (unpublished). However, boys were more parasitized than girls although this result was not statistically significant. Generally speaking, in Mali, there is no gender discrimination with respect to access to water. However, each sex typically has either a domestic or recreational activity that draws them to the water. This result is similar to that reported by Dabo et al. [10, 22], whose study included villages in the same area as those in the present study, for younger children [13]. These results are in contrast to Ezeh et al. [14], who reported that women had a higher prevalence of the disease than their male counterparts.

Our study also revealed a high (not significant) age-related prevalence among primary school students (11–14 years). This is consistent with the findings in Bamako (Mali) (10) and can be attributed to the activities of students in this age group which bring them in frequent contact with the water. In contrast, Angora et al. [15] observed no difference in prevalence between the three age groups studied in a study conducted in Côte d'Ivoire.

Concerning socio-economic parameters, children who used the river/pond as a domestic water source and those who urinated and bathed in the river, were more affected by urinary schistosomiasis, than those who didn’t. This observation is to some extent logical since bilharzia is directly related to exposure to contaminated fresh water. These results are the same as those reported by several authors [23, 24] where the relationship between the prevalence of schistosomiasis and contact with freshwater bodies infested with cercariae is well established. Children with farming parents were the most affected by urinary schistosomiasis. This could be explained by the fact that children commonly work with their parents on the farm. This argument is also illustrated in the report by Ugbomoiko et al. [23], which showed that parents with a higher level of education could better understand preventive strategies and explain them to their children. Our results are supported by Geleta et al. and Angora et al. [15, 25], which show that "farmer" as a parental profession was significantly associated with increased frequency of the disease. However, our findings are in contrast to those reported by Onyekwere et al. [13], where, in Nigeria, no significant association was reported between occupation and infection status. In our study, it is not surprising that the children of trading parents were as infected as children of peasants because in Mali people carry out several activities at the same time and could be traders, fishermen or peasants.

An analysis of clinical signs in children with urinary schistosomiasis showed that 46.1% of children suffer from abdominal pain, 54.0% from pollakiuria, 39.6% from dysuria and 9.3% from hematuria. Our study revealed that only hematuria was associated urogenital schistosomiasis and at intensity. The hematuria rate obtained in our study was lower than that obtained by Keita et al. (25.4%) in Molodo in 2005. Keita et al. also reported a high rate of pollakiuria (19.8%) [26].

Today, ultrasound examination allows for a detailed examination of the pathology of the urinary tract in *S. haematobium* infection and provides a more accurate assessment of internal damage than analyses which involve a parasitological determination of eggs or urinalysis. In addition, ultrasound is an effective method for assessing the course of the damage, as it is safe and more effective especially when combined with parasitological examinations. Although it is used less than it should be, it remains a standard tool in the management of schistosomiasis, and its use is even less in large areas of sub-Saharan Africa where the disease is endemic. Few studies have used it to study the morbidity associated with urinary schistosomiasis. It should be noted that the other limiting factor for this method is cost. The cross-referencing of ultrasound signs and parasitological findings showed that most children with urinary schistosomiasis had a focal lesion (92.6%), diffuse thickening (41.7%), single and multiple masses (8.0%), single and multiple polyps (2.5%) and a probable and very likely score of schistosomiasis (71.8%). These rates are significantly higher than those found by Keita et al. 2005 in Molodo where they observed that an irregular bladder wall was the most frequent lesion with thick walls (2.9%) and 2.3% had bladder masses and 0.9% had bladder polyps. Similarly, Keita et al. reported a rate of 26.6% for bladder wall thickening in the Dogon Plateau and the Office du Niger [26]. Despite the high prevalence of infection, the morbidity was low. This observation was also made by Lanuit et al. [27] in the Richard Toll region of Senegal and also by Alfidja et al. [28]. The low morbidity observed is likely due to the fact that complications appear only after several years of infection. Although ultrasound was less effective for children than for adults, the decision to perform ultrasound on children rather than adults in this study was made to test the hypothesis that the presence of hybrid strains of schistosomes (diagnosed only from eggs excreted by children) might reduce the efficacy of praziquantel, which might then lead to greater morbidity in hybrid parasite carriers compared with non-hybrid carriers. We later realized that another approach was needed to study this correlation, including knowing the precise treatment status of children and ensuring the existence of hybrid strains well before the study. As these conditions were not met, we abandoned this comparison between the presence of hybrids and the frequency of morbid lesions. The various treatment campaigns which have worked to distribute praziquantel in affected areas for a period of more than 10 years (National Program to Combat Schistosomiasis and Helminths) have successfully reduced the rate of complications. The high percentage of symptoms associated with high loads reinforces the idea that further schistosomiasis-related morbidity studies are still needed.

In this work, we were limited by the lack of information on the timing and number of mass distribution campaigns of praziquantel in the area, which impinged on some of the study objectives of the study. We believe that regular mass treatments could further reduce or almost correct the low morbidity rate recorded in this study.

## Conclusions

Our results show that *Schistosoma haematobium* infection was still endemic in the study site despite more than a decade of mass treatment with praziquantel. However, the low rate observed in some localities (Babaroto) probably reflects the positive impact of treatment on infection. Bathing, parents' occupational profile and home water supply were the socioeconomic factors associated with infection. Abdominal pain, dysuria, hematuria, and pollakiuria were the frequently described clinical signs. Ultrasound examinations revealed that focal lesions were recorded in all villages with a low rate in Diakalel. From the ultrasound data, diffuse and focal thickening were observed. Ultrasound signs and parasitological results show that irregularity and thickening were strongly associated with urinary schistosomiasis with a probable urinary schistosomiasis score. The high percentage of symptoms associated with high loads reinforces the idea that further studies in terms of schistosomiasis-related morbidity are still needed.

## Data Availability

Not applicable.
